# Female Mice Lacking LSD1 in Myeloid Cells Are Resistant to Inflammatory Bone Loss

**DOI:** 10.3390/cells14141111

**Published:** 2025-07-19

**Authors:** Kristina Astleford-Hopper, Flavia Saavedra, Peter Bittner-Eddy, Clara Stein, Jennifer Auger, Rachel Clark, Juan E. Abrahante Llorens, Bryce A. Binstadt, Vivek Thumbigere-Math, Kim C. Mansky

**Affiliations:** 1 Oral Biology Graduate Program, University of Minnesota School of Dentistry, Minneapolis, MN 55455, USA; 2 Department of Inflammation and Immunity, Lerner Research Institute, Cleveland Clinic Foundation, Cleveland, OH 44195, USA; 3 Division of Biological Science, Department of Diagnostic and Biological Sciences, University of Minnesota School of Dentistry, Minneapolis, MN 55455, USA; 4 College of Biological Science, University of Minnesota, Minneapolis, MN 55455, USA; 5 Division of Pediatric Rheumatology, Allergy and Immunology, Department of Pediatrics, University of Minnesota Medical School, Minneapolis, MN 55455, USA; 6 Minnesota Supercomputing Institute, University of Minnesota, Minneapolis, MN 55455, USA; 7 Division of Periodontology, Department of Advanced Oral Sciences, Therapeutics University of Maryland School of Dentistry, Baltimore, MD 21201, USA; 8 Division of Orthodontics, Department of Developmental and Surgical Sciences, University of Minnesota School of Dentistry, Minneapolis, MN 55455, USA

**Keywords:** osteoclasts, epigenetics, inflammation, periodontal disease

## Abstract

Osteoclasts, which are derived from myeloid precursors, are essential for physiologic bone remodeling but also mediate pathological bone loss in inflammatory diseases such as periodontitis and rheumatoid arthritis. Lysine-specific demethylase (LSD1/KDM1A) is a histone demethylase that modulates the chromatin landscape via demethylation of H3K4me1/2 and H3K9me1/2, thereby regulating the expression of genes essential for deciding cell fate. We previously demonstrated that myeloid-specific deletion of LSD1 (LSD1LysM-Cre) disrupts osteoclast differentiation, leading to enhanced BV/TV under physiological conditions. In this study, we show that LSD1LysM-Cre female mice are similarly resistant to inflammatory bone loss in both ligature-induced periodontitis and K/BxN serum-transfer arthritis models. Bulk RNA-seq of mandibular-derived preosteoclasts from LSD1LysM-Cre mice with ligature-induced periodontitis revealed the upregulation of genes involved in inflammation, lipid metabolism, and immune response. Notably, LSD1 deletion blocked osteoclastogenesis even under TGF-β and TNF co-stimulation, which is an alternative RANKL-independent differentiation pathway. Upregulation of *Nlrp3*, *Hif1α*, and *Acod1* in LSD1LysM-Cre preosteoclasts suggests that LSD1 is essential for repressing inflammatory and metabolic programs that otherwise hinder osteoclast commitment. These findings establish LSD1 as a critical epigenetic gatekeeper integrating inflammatory and metabolic signals to regulate osteoclast differentiation and bone resorption. Therapeutic inhibition of LSD1 may selectively mitigate inflammatory bone loss while preserving physiological bone remodeling.

## 1. Introduction

Osteoclasts are multinucleated giant cells derived from the monocyte/macrophage lineage that play a pivotal role in skeletal remodeling and repair. Osteoclasts result from the fusion of mononuclear precursors derived from the monocyte/macrophage lineage [1]. Under physiological conditions, osteoclastogenesis is primarily regulated by macrophage colony-stimulating factor (M-CSF) and receptor activator of NF-κB ligand (RANKL), which are secreted by osteoblasts and osteocytes [2]. However, in chronic inflammatory diseases such as periodontitis and rheumatoid arthritis, this regulatory process is subverted: sustained immune activation drives excessive osteoclast differentiation. Innate immune cells infiltrate the synovium or periodontium and secrete proinflammatory cytokines, which facilitate osteoclastogenesis and amplify bone resorption [3]. Tumor necrosis factor (TNF) is a central mediator of both periodontal and joint inflammation [4,5], indirectly enhancing osteoclastogenesis by increasing RANKL expression and stimulating the release of cytokines like IL-1β and IL-6 [6,7,8,9,10,11]. However, TNF alone is insufficient to drive osteoclast differentiation, suggesting a need for additional molecular signaling within the inflamed microenvironment.

Recent studies have demonstrated that TGF-β is capable of priming osteoclast precursors to respond to TNF, which enables TNF-driven osteoclast differentiation independent of RANKL [12]. This alternative pathway has relevance in periodontitis, as elevated levels of TGF-β have been detected in the saliva, serum, and gingival crevicular fluid of periodontitis patients [13,14]. This suggests that TGF-β and TNF may collaborate in driving inflammation-induced osteoclast differentiation [12].

Additionally, emerging evidence suggests that osteoclast precursor populations differ substantially between physiological and inflammatory conditions [15,16]. For example, arthritis-associated osteoclastogenic macrophages (AtoMs), defined by a CX3CR1^+^, Ly6C^int^, F4/80^+^, I-A^+^/I-E^+^ phenotype, exhibit robust osteoclastogenic potential in inflamed joints but do not contribute to physiological bone remodeling [17]. Targeted ablation of AtoMs in experimental arthritis models attenuates bone erosion while preserving normal skeletal remodeling, emphasizing that distinct ontogenies and microenvironmental cues govern osteoclastogenesis in health and disease.

Despite these advances, the epigenetic mechanisms that integrate inflammatory and metabolic signals to regulate osteoclast differentiation remain poorly understood. Lysine-specific demethylase 1 (LSD1/KDM1A) is a histone demethylase that removes methyl groups from H3K4me1/2 and H3K9me1/2, thereby modulating chromatin accessibility and transcriptional programs critical for myeloid-lineage commitment. We previously demonstrated that myeloid-specific deletion of LSD1 in female mice impairs osteoclast differentiation and increases bone volume under physiological conditions and is associated with upregulation of IFN-β-responsive genes [18]. Given that TGF-β downregulates IFN-β targets to enable TNF-mediated osteoclast differentiation [12], we hypothesized that LSD1 may be essential for suppressing IFN-β signaling and permitting osteoclastogenesis under inflammatory conditions.

To test this hypothesis; we examined the consequences of LSD1 deletion in two murine models of inflammatory bone loss: ligature-induced periodontitis (LIP) and K/BxN serum-transfer arthritis. We found that female myeloid-specific LSD1 conditional knockout mice (LSD1cKO) are resistant to bone loss in both models, exhibiting impaired osteoclastogenesis and preserved bone volume. Transcriptomic analyses of mandibular and femur-derived preosteoclasts from LIP LSD1cKO mice revealed upregulation of genes involved in innate immunity, inflammation, lipid transport and metabolism. Notably, LSD1-deficient preosteoclasts failed to differentiate even when co-stimulated with TGF-β and TNF, indicating that LSD1 is indispensable for the epigenetic reprogramming required for osteoclast-lineage commitment in inflammatory settings, acting in part by suppressing IFN-β–responsive genes. Collectively, these findings establish LSD1 as a key epigenetic integrator of inflammatory and metabolic cues that govern osteoclastogenesis and highlights LSD1 as a promising therapeutic target for mitigating inflammatory bone loss.

## 2. Materials and Methods

### 2.1. Ethics

Procedures were reviewed and approved as described previously [18].

### 2.2. LSD1 Mice

The *LSD1* floxed and *LSD1 LysM-Cre* mice used in this study have been previously described, and sources are shown in Table 1 [18].

### 2.3. Primary Osteoclast Culture from Femurs

Osteoclast cultures derived from the bone marrow of the femur were generated as previously described [18].

### 2.4. Primary Osteoclast Culture from Mandibles

Osteoclast cultures derived from the bone marrow of the mandible were generated as previously described [19].

### 2.5. Bulk RNA-SEQ

Bone-marrow cells from the mandible were collected from 3-month-old female Lsd1LysM-WT and Lsd1LysM-cKO mice. The mice were placed in one of two groups: a group that had been subjected to ligature-induced periodontitis for 7 days or a control group with no ligature. Osteoclast-culture generation, RNA isolation, and bulk RNA sequencing were conducted as previously described [18]. The data are available in Geo GSE299905.

### 2.6. Real-Time Quantitative PCR Analysis

Quantitative PCR analysis was conducted as previously described [18]. The forward and reverse primer pairs for each gene are shown in Table 2. Primers to measure expression of *Lsd1*, *Acp5*, *Nfatc1*, *Dc-stamp*, *Ctsk* and *Hprt* have been previously described [18].

### 2.7. Ligature-Induced Periodontitis (LIP)

Six male LSD1WT, five male LSD1cKO, four female LSD1WT, or five female LSD1cKO littermates were anesthetized with 75–100 mg/kg of ketamine and 5–10 mg/kg of xylazine by intraperitoneal injection. All mice were 2 months of age at the time of ligature placement. A 5–0 silk suture (Roboz Surgical, Gaithersburg, MD, USA) was placed with the aid of a stereo-microscope using a method previously described to avoid trauma to the gingival tissue [20]. Ligatures were left in place for 7 days. Ligated mice were co-caged with non-ligated siblings both prior to and following ligature placement. The mice analyzed for bone loss were different from the mice used for gene-expression analysis (N = 4 of each genotype). When cells were collected for gene-expression analysis, ligatures were placed on both the left and right maxillary second molars. The mice were euthanized by CO_2_ inhalation.

### 2.8. Micro-CT Analysis for Alveolar Bone Loss

After 7 days of LIP, the mouse maxillae were dissected and treated as described in [21]. Both the non-ligated and ligated sides of the maxillae were registered three-dimensionally using Data Viewer software (version 1.5.4.0, Bruker-Micro-CT, Kontich, Belgium). The volume of interest (VOI) was determined for the ligated (left second maxillary molar) and non-ligated (right second maxillary molar) regions. Ligated and non-ligated VOIs were imported into CT Analyzer software (version 1.10.1) to determine the bone volume percentage in each mouse. The difference between bone volumes in the VOIs was calculated as the bone volume percentage of the non-ligated VOI minus the bone volume percentage of the ligated VOI.

### 2.9. K/BxN Serum-Transfer Arthritis Mouse Moadel

Arthritis was induced via injection of K/BxN mouse serum [22]. K/BxN serum was collected from K/BxN mice at 8 weeks of age. Three-month-old LSD1WT and LSD1LysM-cKO female mice (N = 5 of each genotype) were induced via intraperitoneal injection of 150 µL of K/BxN serum on days 0 and 3. The mice were observed, and ankle thickness and clinical scores were measured every day for 11 days. Clinical scores ranged from 0 (no arthritis) to 3 (maximum arthritis). Mice were sacrificed on day 11 by CO_2_ inhalation, and ankles and tibiae were harvested for histological and μCT analyses.

### 2.10. Micro-CT Analysis for Quantification of Rheumatoid Arthritis-Induced Boney Lesions

The tibiae and ankles from three-month-old mice treated with K/BxN serum were isolated, wrapped in gauze, and stored in PBS at −80 °C. At the time of scanning, the tibiae were defrosted to room temperature and scanned in PBS with a 1 mm aluminum filter using a XT H 225 micro-computed tomography machine (Nikon, Tokyo, Japan) at listed voxel size for each scan. Scan settings were set to 120 kV, 61 μA, 720 projections, two frames per projection, and an integration time of 708 milliseconds. Three-dimensional reconstruction volumes were calculated for each scan using CT Pro 3D. These 3D volumes were then converted to bitmap datasets using VG Studio MAX 3.2. Before analysis, the scans were rotated using DataViewer, and CT Analyzer was used to perform morphometric analyses, as previously described [23].

### 2.11. Paraffin-Embedded TRAP Staining

The tibiae and ankles or maxillae from three-month-old mice were isolated after micro-CT, fixed in Z-fix (Anatech LTD, Battlecreek, MI, USA, catalog #NC9378601), placed in 10% EDTA (pH 7.4) for decalcification, then paraffin-embedded and sectioned for histological staining. Bone sections were processed for TRAP staining, as previously described [18]. Images were taken using light microscopy and analyzed.

### 2.12. TGF-β- and TNF-Induced Osteoclast Differentiation

Bone marrow from LSD1WT or LSD1cKO mice was isolated as described above. Femoral or mandibular cells were plated on osteoclast media supplemented with one of two treatments: 1.5% CMG14–12 conditioned media (TGFβ group) or 1.5% CMG14–12 conditioned media with TGF-β (1 ng/mL) (TGFβ+ group). The cells were incubated for 48 h at 37 °C to allow them to differentiate into bone-marrow macrophages (Day 0). The TGFβ- and TGFβ+ groups were stimulated with CMG14–12 conditioned media and TNF (40 ng/mL). Cells were harvested on day 2 for RT-qPCR analysis.

### 2.13. Statistical Analysis

Statistical analysis was carried out as previously described [18].

## 3. Results

### 3.1. Deletion of LSD1 Inhibits LIP-Induced Alveolar Bone Loss

**Figure 1:**
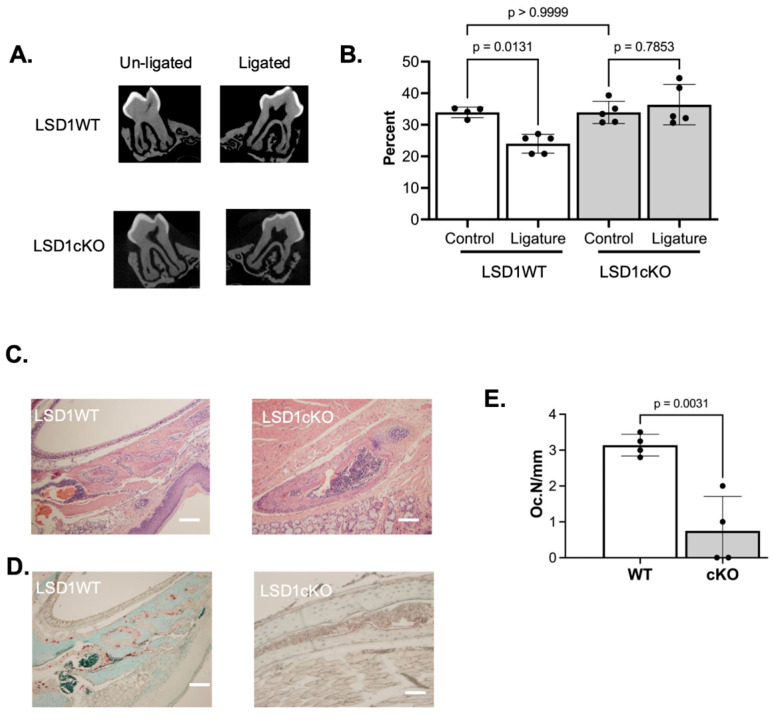
Female LSD1cKO mice are resistant to LIP-induced alveolar bone loss. (**A**) Representative micro-CT image. (**B**) Bone volume relative to total volume (%) in control and LIP-induced periodontitis in female LSD1WT (n = 4) and LSD1cKO mice (n = 5). Samples were compared using one-way ANOVA followed by Tukey’s post-hoc test. (**C**) Representative image of 20× H and E-stained maxillae. (**D**) Representative 20× TRAP-stained maxillae. (**E**) Number of osteoclasts per mm. Scale bar = 1 mm. Numbers of osteoclasts were compared using Student’s *t*-test.

To investigate the role of LSD1 in periodontitis-mediated inflammatory bone loss, silk ligatures were placed around the maxillary second molar in 2-month-old LSD1WT and LSD1cKO male and female mice for 7 days to induce localized inflammation. Micro-CT analysis revealed a reduction of ~30% in bone-volume fraction (BV/TV) in female LSD1WT mice; however, female LSD1cKO mice showed no significant changes in BV/TV (Figure 1A,B, *p* = 0.0131 for LSD1WT and *p* = 0.7853 for LSD1cKO). In contrast, both male LSD1WT and male LSD1cKO mice exhibited a reduction of ~15% in BV/TV following ligature placement (Supplementary Figure S1A,B, *p* < 0.001 for LSD1WT and *p* = 0.008 for LSD1cKO), suggesting a sex-dependent phenotype. Histological analysis confirmed a significant reduction in osteoclast numbers in the alveolar bone of female LSD1cKO mice compared to LSD1WT controls, whereas no significant difference in osteoclast number was observed between genotypes among male mice (Figure 1D,E, Supplementary Figure S1D,E). These findings demonstrate that LSD1 expression is required for osteoclast-mediated alveolar bone loss in females under inflammatory conditions and suggest that LSD1 contributes to sex-specific regulation of osteoclastogenesis during periodontal inflammation.

### 3.2. Knockout of LSD1 Results in Decreased Bone Lesions and Ankle Thickness in Mice with K/BxN-Serum-Transferred Arthritis

**Figure 2:**
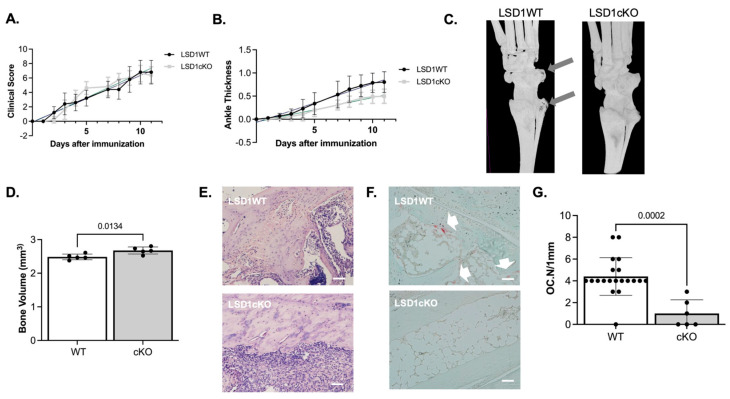
Arthritis-induced osteoclast differentiation is minimal in female LSD1cKO mice. K/BxN-serum-transferred arthritis in female LSD1WT (n = 5) and LSD1cKO (n = 5) mice. LSD1WT mice are represented by the black circles, and LSD1cKO mice are represented by gray squares. (**A**) Clinical score and (**B**) ankle thickness. Samples were compared using a paired Student’s *t*-test. (**C**) Representative micro-CT images. Gray arrows indicate areas of bone erosion. (**D**) Bone volumes of LSD1WT (n = 5) and LSD1cKO (n = 5) mice. (**E**) 20× representative H and E and (**F**) 20× TRAP images and (**G**) number of osteoclasts per mm of bone. White arrows indicate TRAP-positive cells. Scale bar = 1 mm. Bone volumes and numbers of osteoclasts were compared using Student’s *t*-test.

To determine if the protective effects of LSD1 deletion extend beyond periodontal inflammation, we used the K/BxN serum-transfer arthritis model, examining mouse ankle thicknesses and changes in bone volume. Based on our findings from the LIP model, only female LSD1WT and LSD1cKO mice were assessed, as protection from bone loss was observed exclusively in females. Neither clinical arthritis scores nor ankle swelling differed between the LSD1WT and LSD1cKO mice (Figure 2A,B). On day 11, micro-CT analysis of the ankles revealed a significant increase in bone volume in LSD1cKO mice compared to LSD1WT mice (Figure 2C, *p* = 0.0134). Histological evaluation further confirmed a significant reduction in osteoclast numbers in the inflamed joints of LSD1cKO mice (Figure 2D,F, *p* = 0.0002), indicating that LSD1 is required for osteoclastogenesis and subsequent bone erosion in this inflammatory context. Collectively, these findings mirror the phenotype observed in the LIP model and reinforce that LSD1-dependent osteoclast differentiation is a central driver of bone loss across anatomically and mechanistically distinct models of inflammation.

### 3.3. Increase in IFN-β Regulated Genes in LSD1cKO Preosteoclasts

**Figure 3:**
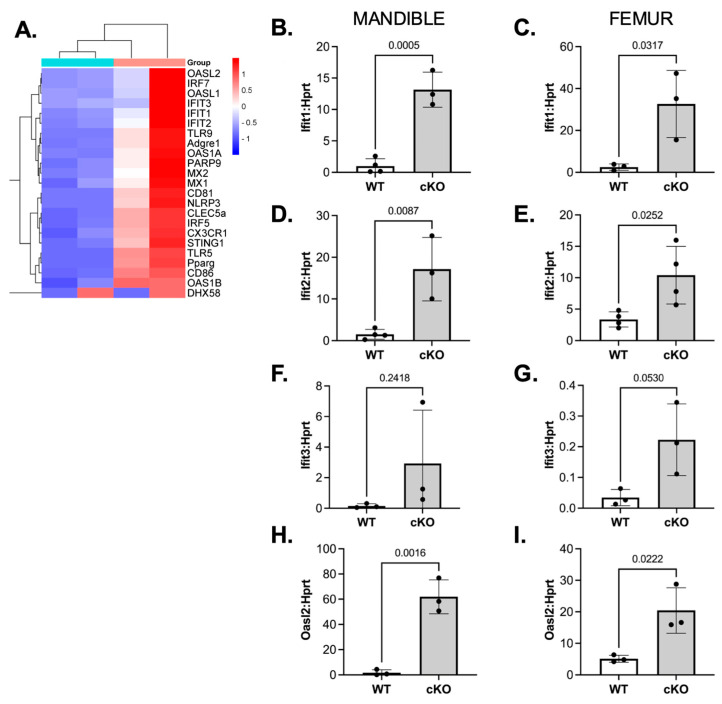
LSD1cKO mice have increased expression of inflammatory genes in osteoclasts from ligated mice. (**A**) Heat map of inflammation-related genes. LSD1WT = green tissue-cluster heading and LSD1cKO = pink tissue-cluster heading. (**B**–**I**) Mandibular and femur-derived bone-marrow cells were isolated from female LSD1WT and LSD1cKO mice. Bone-marrow cells were cultured in M-CSF and RANKL for 2 days. RNA was isolated, and qRT-PCR was performed to analyze gene expression. Verification of upregulated genes: (**B**,**C**) is *Ifit1*, (**D**,**E**) is *Ifit2*, (**F**,**G**) is *Ifit3*, and (**H**,**I**) is *Oasl2*. (**B**,**D**,**F**,**H**) are from mandibular-derived preosteoclasts, and (**C**,**E**,**G**,**I**) are from femur-derived preosteoclasts.

Given that LSD1cKO mice were resistant to inflammatory bone loss, we next examined transcriptional changes in osteoclast precursors to identify potential molecular mechanisms underlying this phenomenon. Bulk RNA-SEQ was performed on mandibular-bone-marrow-derived osteoclast precursors isolated from female LSD1WT and LSD1cKO mice subjected to 7 days of LIP. The bone marrow was flushed, and osteoclast precursors were incubated with M-CSF and RANKL for 2 days prior to RNA extraction and bulk RNA sequencing. Using DAVID analysis of differentially expressed genes (DEGs), we determined up- and down-regulated pathways in LSD1cKO versus LSD1WT mandibular-derived osteoclasts (Figure 3A). Genes involved in inflammation and immune-related pathways were markedly upregulated in LSD1cKO preosteoclasts (Figure 3A). To further investigate whether these changes were consistent across skeletal sites, we performed qRT-PCR for canonical IFN-β–regulated genes in both mandibular and femur-derived preosteoclasts from LIP LSD1WT and LSD1cKO mice. IFN-β related genes (*Ifit1*, *Ifit2*, *Ifit3*, and *Oasl2*) were significantly upregulated at both sites, with a notably higher-fold increase observed in mandibular-derived cells (Figure 3B–H). Interestingly, even in the LSD1WT mice, femur-derived preosteoclasts exhibited a higher baseline expression of IFN-β–responsive genes compared to their mandibular counterparts, suggesting site-specific differences in IFN-β signaling and/or osteoclastogenic potential. This may be due to the inflammatory stimulus being present in the mandible or an enhancement of osteoclast differentiation in mandibular-derived cells [19]. In combination, these results extend our prior findings and confirm that LSD1 is essential for the repression of IFN-β–responsive genes in osteoclast precursors. Loss of LSD1 leads to sustained activation of this anti-osteoclastogenic pathway, thereby impairing osteoclast differentiation under inflammatory conditions [18].

### 3.4. LSD1 Expression in the Femur Is Necessary for TNF-Induced Osteoclast Differentiation

**Figure 4:**
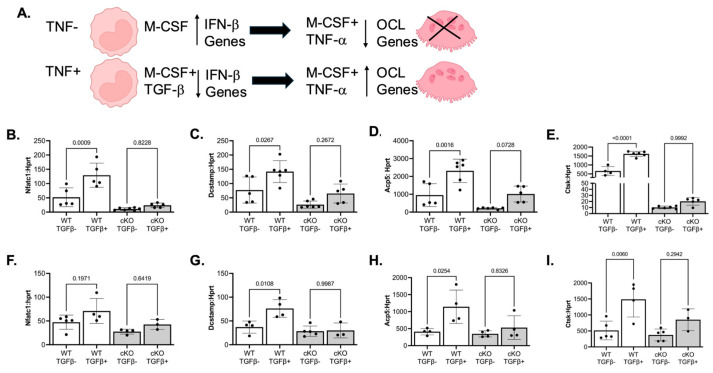
LSD1 expression in the femur and mandible is necessary for TNF-induced osteoclast gene expression. (**A**) Mechanistic model by which TNF and TGF-β induce osteoclast precursors to differentiate based on the model published by Xia et al. [12]. (**B**–**E**) qRT-PCR of osteoclast genes from femur-derived osteoclasts; (**F**–**I**) qRT-PCR of osteoclast genes from mandibular-derived osteoclasts. Data shown are from at least three independent experiments. Data are graphed relative to *Hprt.* Samples were compared using one-way ANOVA followed by Tukey’s post-hoc test.

To explore whether LSD1 is functionally required for inflammatory osteoclastogenesis via non-canonical cytokine signaling, we evaluated its role in the RANKL-independent differentiation pathway driven by TGF-β and TNF. Recent studies have shown that TGF-β priming of osteoclast precursor cells prior to the addition of TNF downregulates IFN-β–responsive genes, thereby permitting TNF-induced osteoclast differentiation in the absence of RANKL [12,24] (Figure 4A). Given our observation of elevated IFN-β–regulated transcripts in LSD1cKO mandibular and femoral preosteoclasts (Figure 3), we hypothesized that LSD1 may be essential for this epigenetic silencing step. To test this hypothesis, mandibular and femur-derived preosteoclasts from female LSD1WT and LSD1cKO mice were primed with TGF-β and then stimulated with TNF without RANKL. In LSD1WT preosteoclasts from the femur (Figure 4B–E) and mandible (Figure 4F–I), this cytokine sequence induced robust expression of key osteoclast genes (*Nfatc1*, *Dcstamp*, *Acp5*, and *Ctsk*), a response consistent with effective differentiation. In contrast, LSD1cKO cells failed to upregulate these genes under identical conditions, regardless of skeletal origin. These findings demonstrate that LSD1 is indispensable for TGF-β/TNF-mediated osteoclastogenesis and that its absence locks osteoclast precursors in a non-permissive transcriptional state, likely through failure to repress IFN-β–driven inhibitory programs. Future studies will include measurement of inflammatory cytokines such as TNF and TGF-β in the oral mucosa and bone marrow to determine whether this RANKL-independent mechanism of osteoclast differentiation is responsible for bone loss in LIP.

### 3.5. LSD1cKO Preosteoclasts Have Dysregulated Inflammatory Genes

**Figure 5:**
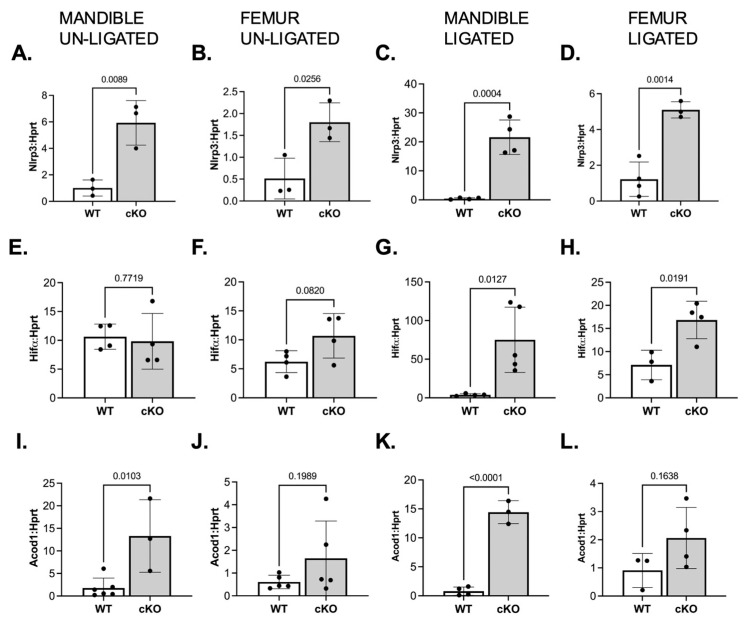
Unligated and ligated LSD1cKO mice have upregulated expression of inflammatory genes. RNA was isolated from mandibular and femur-derived osteoclasts isolated from female LSD1WT and LSD1cKO mice that remained unligated or that had been ligated for 7 days. Bone-marrow cells were incubated with M-CSF and RANKL for 2 days. qRT-PCR was performed to analyze gene expression. (**A**–**D**) is *Nlrp3*, (**E**–**H**) is *Hif1a*, and (**I**–**L**) is *Acod1*. Graphs (**A**,**C**,**E**,**G**,**I**,**K**) represent mandibular-derived preosteoclasts, and graphs (**B**,**D**,**F**,**H**,**J**,**L**) represent femur-derived preosteoclasts. (**A**,**B**,**E**,**F**,**I**,**J**) represent cells isolated from unligated mice, and (**C**,**D**,**G**,**H**,**K**,**L**) represent cells isolated from ligated mice. Data shown are from at least three independent experiments. Data are graphed relative to *Hprt.* Samples were compared using an unpaired Student’s *t*-test.

To further dissect the inflammatory transcriptional landscape of LSD1-deficient preosteoclasts, we evaluated the expression of genes associated with innate immune activation and metabolic adaptation during inflammation. qRT-PCR analyses were performed on mandibular and femur-derived preosteoclasts from both ligated and unligated LSD1WT and LSD1cKO female mice. Notably, the inflammasome component *Nlrp3* [25] was significantly upregulated in LSD1cKO preosteoclasts from both skeletal sites even in the absence of ligature placement (Figure 5A,B), indicating a basal increase in inflammasome priming.

First, we investigated the expression of *Hif1a*, a hypoxia-inducible transcription factor known to enhance osteoclast survival and function under inflammatory stress. In our mice, *Hif1a* was unchanged in unligated mice (Figure 5E,F) but significantly elevated in both mandibular and femur-derived cells following ligature placement in LSD1cKO mice (Figure 5G,H). Similarly, *Acod1*, which encodes the mitochondrial enzyme itaconate synthase and is associated with immune-metabolic reprogramming in inflammatory macrophages [26,27,28], was upregulated in both mandibular and femur-derived LSD1cKO preosteoclasts under basal conditions (Figure 5I,J), and remained selectively elevated in the mandible after ligature (Figure 5K,L). These data indicate that LSD1 deficiency results in inappropriate activation of proinflammatory and metabolic pathways in osteoclast precursors, features consistent with an inflammatory macrophage-like phenotype. The persistent expression of *Nlrp3*, *Hif1a*, and *Acod1* suggests that LSD1 normally constrains inflammatory transcriptional circuits that, if unchecked, impair osteoclast-lineage commitment under stress conditions.

### 3.6. Loss of LSD1 Expression Enhances Lipid Metabolism and Expression of Transport Genes 

**Figure 6:**
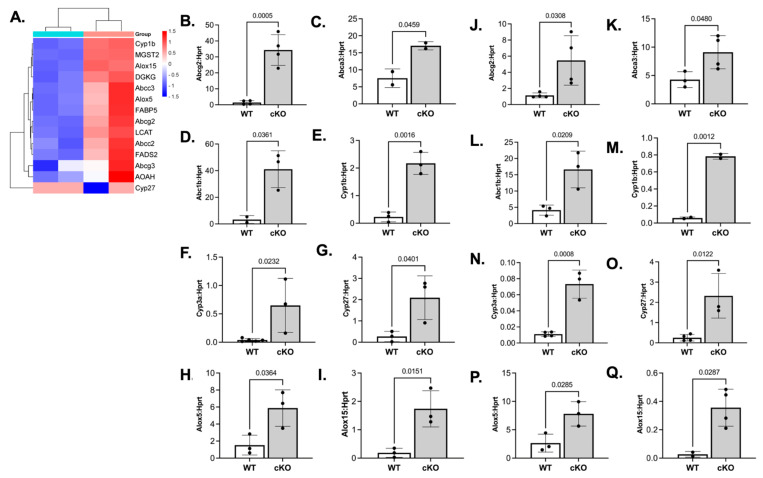
LSD1cKO mice have increased expression of genes associated with lipid metabolism and transport in preosteoclasts from ligated mice. (**A**) Heat map of genes associated with lipid metabolism and transport. LSD1WT = green tissue-cluster heading and LSD1cKO = pink tissue-cluster heading. (**B**–**Q**) RNA was isolated from mandibular or femur-derived preosteoclasts that had been incubated in M-CSF and RANKL for 2 days. Mandibular and femur-derived bone-marrow cells were isolated from female LSD1WT and LSD1cKO mice. qRT-PCR was performed to analyze gene expression. Verification of upregulated genes: (**B**,**J**) is *Abcg2*, (**C**,**K**) is *Abca3*, (**D**,**L**) is *Abc1b*, (**E**,**M**) is *Cyp1b*, (**F**,**N**) is *Cyp3a*, (**G**,**O**) is *Cyp27*, (**K**,**P**) is *Alox5*, and (**I**,**Q**) is *Alox15*. Graphs (**B**–**I**) represent mandibular preosteoclasts, and graphs (**J**–**Q**) represent femur-derived preosteoclasts. Data shown are from at least three independent experiments. Data are graphed relative to *Hprt.* Samples were compared using an unpaired Student’s *t*-test.

Beyond dysregulation of inflammatory genes, transcriptomic analysis of mandibular and femur-derived preosteoclasts from LIP LSD1cKO mice also revealed enrichment of pathways related to lipid metabolism and transport (Figure 6A–Q). To validate these findings and assess skeletal-site consistency, qRT-PCR was performed on both mandibular and femur-derived preosteoclasts. Several genes involved in cholesterol transport (*Abcg2*, *Abca3*, *Abcb1b*), cytochrome P450 family members (*Cyp1b1*, *Cyp3a11*, *Cyp27a1*), and lipid oxidation pathways (*Alox5*, *Alox15*) were significantly upregulated in LSD1cKO preosteoclasts following LIP inflammation (Figure 6B–Q). Although these changes were consistently observed across skeletal sites, mandibular-derived cells generally exhibited higher-fold changes. These findings suggest that LSD1 plays a role in restricting lipid-metabolism programs that are aberrantly activated during inflammation. Elevated expression of lipid-handling and oxidation-related genes in LSD1cKO preosteoclasts may reflect a shift toward a macrophage-like immune-metabolic phenotype uncoupled from the osteoclast-differentiation trajectory. Taken together, our data indicate that LSD1 coordinates the transcriptional balance between inflammatory activation, lipid metabolism, and osteoclast-lineage commitment and that its loss disrupts this balance in a way that impairs pathological bone resorption without inhibiting induction of inflammatory genes.

## 4. Discussion

In this study, we demonstrate that conditional deletion of LSD1 in the myeloid lineage confers protection against inflammatory bone loss in two distinct murine models of osteolytic disease: LIP and K/BxN serum-transfer arthritis. Utilizing the LIP model, a well-established model for dissecting host immune contributions to periodontal bone resorption [29], we observed that female LSD1cKO mice were protected from alveolar bone loss, while their LSD1WT counterparts exhibited a ~30% reduction in bone-volume fraction. Notably, this protection was sex-specific, as male LSD1cKO mice showed no significant resistance to LIP-induced bone loss, mirroring our prior physiological findings [18]. To the best of our knowledge, this is the first report of a sex-specific resistance to LIP-induced bone loss. To extend these observations to an independent inflammatory context, we employed the K/BxN serum-transfer model of rheumatoid arthritis. Consistent with the LIP data, female LSD1cKO mice exhibited preserved trabecular bone volume and markedly fewer osteoclasts in the arthritic joints despite clinical scores and degrees of ankle swelling to comparable to those of LSD1WT mice. Together, these findings establish that LSD1 is essential for osteoclast-mediated bone resorption in response to inflammatory stimuli and suggest that LSD1 inhibition may offer a sex-specific therapeutic strategy for preventing skeletal damage in chronic inflammatory diseases such as periodontitis and rheumatoid arthritis.

The observed sex-specific phenotype raises important questions about the interaction between epigenetic regulation and sex hormones in osteoclast biology. Estrogen is a well-established modulator of bone turnover and inflammatory signaling, exerting suppressive effects on both osteoclast differentiation and cytokine production [30,31,32,33]. Prior studies have shown that estrogen-receptor signaling influences the transcriptional landscape of myeloid cells and modulates the expression of genes involved in osteoimmune responses [34,35]. It is plausible that in female mice, estrogen may synergize with LSD1 loss to amplify repression of osteoclastogenic programs, or, alternatively, that estrogen deficiency in males may blunt the impact of LSD1 deletion on inflammatory osteoclastogenesis. Additionally, LSD1 itself has been implicated in signaling pathways linked to sex-hormone receptors in other tissues, raising the possibility of a mechanistic link between epigenetic and hormonal regulation in osteoclast precursors [36,37,38]. Further investigation into how estrogen, androgen, and LSD1 signaling converge in bone-resorbing myeloid cells will be critical for understanding the basis of this sexual dimorphism and for translating LSD1-targeted therapies across sexes.

To elucidate the molecular pathways underlying the protective effects in LSD1cKO mice, we performed bulk RNA sequencing of mandibular-derived osteoclast precursors from LIP mice. We noted significant enrichment of genes involved in inflammatory signaling, lipid transport, and metabolic adaptation, pathways known to shape immune-cell fate and function [39]. Notably, the upregulated genes included markers associated with M1-like proinflammatory macrophage polarization, such as members of the cytochrome P450 (*Cyp*), ATP-binding cassette transporter (*Abc*), and arachidonate lipoxygenase (*Alox*) gene families. These transcriptional signatures suggest that in the absence of LSD1, osteoclast precursors may be redirected toward a proinflammatory macrophage-like state rather than progressing along the osteoclast-differentiation trajectory. Given that lipid metabolism and inflammatory signaling are tightly coupled in macrophage biology, our findings raise the possibility that LSD1 serves as a chromatin-based switch that represses myeloid inflammatory programs while enabling osteoclastogenic gene expression. Future investigations are warranted to determine whether LSD1 directly represses these inflammatory circuits or acts via secondary regulators. Moreover, studies examining cytokine secretion and functional polarization of LSD1-deficient monocytes will provide critical insights into how lineage bifurcation is epigenetically enforced in the bone microenvironment during inflammation.

In addition to upregulation of IFN-β signaling and lipid metabolism and transport, LSD1cKO preosteoclasts from both mandibular and femoral origins also exhibited elevated expression of key immune-metabolic regulators including *Acod1*, *Nlrp3*, and *Hif1a*. These genes are functionally linked to inflammatory osteoclastogenesis and immune-cell reprogramming. *Acod1* encodes the mitochondrial enzyme aconitate decarboxylase, which produces the anti-inflammatory metabolite itaconate—a molecule that restrains osteoclast differentiation and mediates responses to metabolic stress [28,40,41,42]. NLRP3, a core component of the inflammasome, is critical for IL-1β processing and osteoclast activation. Mice lacking *Nlrp3* exhibit diminished bone resorption and osteoclast numbers in LIP, implicating it as a central mediator of inflammatory bone loss [43]. *HIF1a*, which encodes the oxygen-sensitive transcription factor HIF-1α, has been shown to promote osteoclast differentiation under hypoxic or inflammatory conditions, and its deletion protects against periodontal bone loss [44]. Our data demonstrate coordinated upregulation of these genes in LSD1-deficient preosteoclasts, suggesting that LSD1 normally constrains a transcriptional axis that integrates innate immunity, metabolic adaptation, and osteoclastogenic potential. While it remains to be determined whether these targets are directly regulated by LSD1 at the chromatin level, their consistent elevation across skeletal sites and inflammatory models underscores the broader role of LSD1 as a transcriptional gatekeeper in the osteoimmune interface.

The role of LSD1 in osteoclastogenesis may also intersect with emerging paradigms in innate immune memory, particularly in the context of “trained innate immunity”—a process by which innate immune cells undergo long-lasting epigenetic reprogramming in response to microbial or inflammatory stimuli. While trained innate immunity is protective against infections and tumors, its maladaptive activation has been implicated in chronic inflammatory diseases, including periodontitis and rheumatoid arthritis. A recent study by Li et al. demonstrated that maladaptive trained innate immunity contributes to shared inflammatory comorbidities, such as periodontitis and arthritis, by expanding pathogenic myeloid populations with epigenetically primed phenotypes. Notably, LSD1 (KDM1A) expression was found to be upregulated, implicating it in the chromatin-remodeling events that mediate trained innate responses. Although traditionally viewed as a mediator of histone demethylation during lineage commitment, our findings suggest that LSD1 may also regulate the durability and specificity of inflammatory memory in osteoclast precursors. Given recent evidence that osteoclasts themselves can undergo innate immune training, it is plausible that LSD1 serves as a molecular fulcrum controlling whether inflammatory signals are transiently integrated or epigenetically embedded in the osteoclast lineage. Future studies will be essential to dissect how LSD1 coordinates gene silencing versus activation during successive inflammatory insults and whether targeting LSD1 activity using FDA approved inhibitors can reverse maladaptive osteoimmune memory in the context of chronic disease.

## 5. Conclusions

Our findings establish LSD1 as a pivotal epigenetic regulator of osteoclastogenesis under inflammatory conditions. By repressing anti-osteoclastogenic pathways and facilitating inflammatory-lineage commitment, LSD1 plays a central role in mediating pathological bone loss. Targeting LSD1 may thus offer a novel therapeutic strategy to mitigate pathological bone loss in chronic inflammatory diseases without compromising physiological bone turnover. Importantly, the observed sex-specific protection in LSD1-deficient mice suggests a potential interaction between epigenetic and hormonal signaling pathways. Our ongoing work are aimed at identifying the direct transcriptional targets of LSD1, elucidating its role in epigenetic memory formation within osteoclast precursors, and uncovering the mechanistic basis of its sex-dependent effects on osteoclast differentiation and bone homeostasis.

## Figures and Tables

**Table 1:** Sources of mice and genotyping primers.

Mouse Model	Source and Catalog Number	Providing Laboratory
LSD1 floxed	Jackson Labs 023969	Dr. Stuart Orkin
LysM-Cre	Jackson Labs 026861	none
C57Bl/6J	Jackson Labs 000664	none
**Genotyping primers**		
*LSD1*	F:GCTGGATTGAGTTGGTTGTG	
	R:CTGCTCCTGAAAGACCTGCT	
*LysM-Cre*	F:TCCAATTTACTGACCGTACACCAA	
	R:CCTGATCCTGGCAATTTCGGCTA	

**Table 2:** RT-qPCR primers.

Gene	5′–3′ Primer
*Cyp1b*	F: GCCACTATTACGGACATCTTCGG R: ACAACCTGGTCCAACTCAGCCT
*Cyp27*	F: TCAGGAGACCATCGGCACCTT R: CCAGTCACTTCCTTGTGCAAGG
*Cyp39*	F: ATCCAAAAGATGGCTCCTGGC R: TGTTTCCGTCTCCACCACTTCC
*Abcg2*	F: CAGTTCTCAGCAGCTCTTCGA R: TCCTCCAGAGATGCCACGGAT
*Abcg3*	F: CTTCATGGACGAAGCTGACCTG R: GTGCGGTTCTTTTACCAGCGTC
*Alox5*	F: TCTTCCTGGCACGACTTTGCTG R: GCAGCCATTCAGGAACTGGTAG
*Alox15*	F: GACACTTGGTGGCTGAGGTCTT R: TCTCTGAGATCAGGTCGCTCCT
*Ifit1*	F: CAACTGAGGACATCCCGAAACA R: ATGTGGGCCTCAGTTTCAAAGT
*Ifit2*	F: AGTACAACGAGTAAGGAGTCACT R: AGGCCAGTATGTTGCACATGG
*Ifit3*	F: TGAGGAAGGGTGGACACAAC R: ACATCGCAATTGCCAGTCCA
*Oasl2*	F: AGGGGACAACCCTGAACCA R: TAGGCCAGGCTTCTGCTACA
*Nlrp3*	F: TCACAACTCGCCCAAGGAGGAA R: AAGAGACCACGGCAGAAGCTAG
*Acod1*	F: GGCACAGAAGTGTTCCATAAAGT R: GAGGCAGGGCTTCCGATA
*Hif1a*	F: CCTGCACTGAATCAAGAGGTTGC R: CCATCAGAAGGACTTGCTGGCT

## Data Availability

Bulk RNA SEQ data is available in NCBI Geo GSE299905. RAW PCR data from gene expression is available upon request.

## Supplementary Materials

The following supporting information can be downloaded at: https://www.mdpi.com/article/10.3390/cells14141111/s1. Figure S1: Male LSD1cKO mice have bone loss associated with LIP. (A) Representative micro-CT image (B) bone volume percentage relative to total volume in control and ligature induced periodontitis male LSD1WT (n = 6) and LSD1cKO mice (n = 5). Samples were compared using one-way ANOVA followed by Tukey post-hoc test. (C) Representative image of 20× H and E-stained maxillae (D) Representative 20× TRAP-stained maxillae (E) number of osteoclasts per mm. Scale bar = 1 mm Number of osteoclasts were compared using Student’s *t*-test.
