# Supplementary material for: Female Mice Lacking LSD1 in Myeloid Cells Are Resistant to Inflammatory Bone Loss
Source: Cells. 2025 Jul 19;14(14):1111. doi: 10.3390/cells14141111 (PMC12293761; doi:10.3390/cells14141111)
Supplement: Supplementary file 1 [file cells-14-01111-s001.zip › cells-3733789-supplementary.pdf]

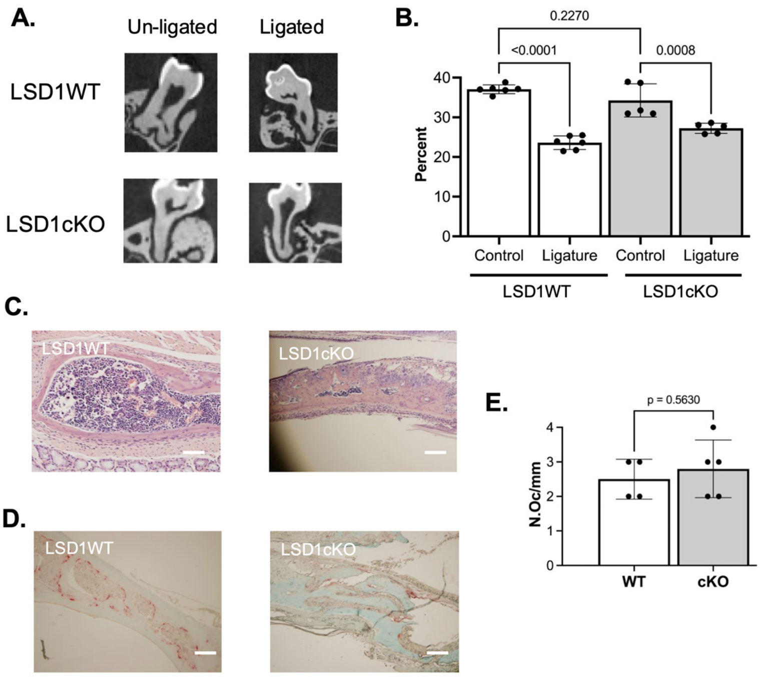

Figure S1. Male LSD1cKO mice have bone loss associated with LIP. (**A**) Representative micro-CT image (**B**) bone volume percentage relative to total volume in control and ligature induced periodontitis male LSD1WT (n = 6) and LSD1cKO mice (n = 5). Samples were compared using one-way ANOVA followed by Tukey post-hoc test. (**C**) Representative image of 20 × H and E-stained maxillae (**D**) Representative 20× TRAP-stained maxillae (**E**) number of osteoclasts per mm. Scale bar = 1 mm Number of osteoclasts were compared using Student's *t*-test.
